# EEG functional brain connectivity strengthens with age during attentional processing to faces in children

**DOI:** 10.3389/fnetp.2022.890906

**Published:** 2022-10-13

**Authors:** Julieta Ramos-Loyo, Paola V. Olguín-Rodríguez, Sara E. Espinosa-Denenea, Luis A. Llamas-Alonso, Sergio Rivera-Tello, Markus F. Müller

**Affiliations:** ^1^ Instituto de Neurociencias, Universidad de Guadalajara, Guadalajara, Jalisco, México; ^2^ Instituto de Ciencias Nucleares, Universidad Nacional Autónoma de México, Ciudad de México, México; ^3^ Centro de Ciencias de La Complejidad, Universidad Nacional Autónoma de México, Ciudad de México, México; ^4^ Universidad Autónoma de Baja California, Ensenada, México; ^5^ Centro de Investigación en Ciencias, Universidad Autónoma del Estado de Morelos, Cuernavaca, Morelos, México; ^6^ Centro Internacional de Ciencias A. C., Cuernavaca, Morelos, México

**Keywords:** functional connectivity, neural networks, EEG, children, age, attention, face processing

## Abstract

Studying functional connectivity may generate clues to the maturational changes that occur in children, expressed by the dynamical organization of the functional network assessed by electroencephalographic recordings (EEG). In the present study, we compared the EEG functional connectivity pattern estimated by linear cross-correlations of the electrical brain activity of three groups of children (6, 8, and 10 years of age) while performing *odd-ball* tasks containing facial stimuli that are chosen considering their importance in socioemotional contexts in everyday life. On the first task, the children were asked to identify the sex of faces, on the second, the instruction was to identify the happy expressions of the faces. We estimated the stable correlation pattern (SCP) by the average cross-correlation matrix obtained separately for the resting state and the task conditions and quantified the similarity of these average matrices comparing the different conditions. The accuracy improved with higher age. Although the topology of the SCPs showed high similarity across all ages, the two older groups showed a higher correlation between regions associated with the attentional and face processing networks compared to the youngest group. Only in the youngest group, the similarity metric decreased during the sex condition. In general, correlation values strengthened with age and during task performance compared to rest. Our findings indicate that there is a spatially extended stable brain network organization in children like that reported in adults. Lower similarity scores between several regions in the youngest children might indicate a lesser ability to cope with tasks. The brain regions associated with the attention and face networks presented higher synchronization across regions with increasing age, modulated by task demands.

## Introduction

The brain is a hyperconnected structure, enclosing anatomically and functionally organized networks. Its intrinsic connectivity patterns can be reconfigured dynamically and adaptively as a consequence of environmental demands (Cohen & D'Esposito, 2016) and may suffer maturational changes at the functional (Smit et al., 2012; Qin et al., 2015) and structural level (Hagmann et al., 2010; Fan et al., 2011).

A close relationship between structural and functional connectivity has been proposed, although there is no perfect match because function reflects complex multisynaptic interactions in structural networks (Park & Friston, 2013; Suárez et al., 2020). Suárez et al. (2020) remark that functional connectivity at rest is thought to reflect the spontaneous neural activity of a finely orchestrated spatio-temporal pattern, which should be reproducible like those observed during tasks performance which patterns are highly organized, reproducible, and comparable with tasks-driven activation patterns. Most of the studies addressing the structural and functional brain connectivity relationships have been conducted using functional magnetic resonance (fMRI) (Park & Friston, 2013; Suárez et al., 2020). Nevertheless, some related work has also been performed using brain electrical activity (EEG) (Chu et al., 2015). Moreover, Arzate-Mena et al. (2022) encountered a straight relationship between the EEG stable correlation pattern (SCP) and the fMRI resting-state network that reflects different time expressions of the same brain activity.

EEG has proven useful to study functional connectivity, as its fine temporal resolution allows the assessment of fast dynamic processes. Different approaches have emerged to extract brain dynamics information from EEG (Pereda et al., 2005; Ansari-Asl et al., 2006; Boccaletti et al., 2006; Bullmore & Sporns, 2009; Bakhshayesh et al., 2019). However, even when highly nonlinear systems are under consideration, linear measures may perform equally well or even better than nonlinear techniques (Mormann et al., 2005; Ansari-Asl et al., 2006; Bakhshayesh et al., 2019), which seems particularly true for the analysis of EEG-signals (Mormann et al., 2005; Müller et al., 2011; Bakhshayesh et al., 2019). Therefore, in the present study, we focus on linear cross-correlations to estimate functional networks.

Regarding linear cross-correlation between selected electrodes located in different scalp regions, Corsi-Cabrera et al. (1997, 2007) observed high within-subject stability in repeated measures across weeks and months in healthy women. In the studies of Müller et al. (2014) and Olguín-Rodríguez et al. (2018), a well-pronounced average cross-correlation pattern was found that spans over the whole scalp. This pattern seems to be independent from the physiological state of a subject like different sleep stages, or awake with open or closed eyes, and remains stable even during a peri-ictal transition of a focal onset seizure. Moreover, this correlation structure seems to have a universal character, since it shows notably high similarity also between subjects. Undoubtedly, finding stationary patterns in otherwise highly nonstationary multivariate data is an important topic, as it can give us insight into the main mechanisms controlling the dynamics of a complex system such as the human brain.

The present study was motivated by the hypothesis articulated by Olguín-Rodríguez et al. (2018) that the SCP reflects the necessarily correlated ongoing brain activity whose one of its principal functions consists in maintaining the brain in an optimal dynamical mode for information processing such that deviations or fluctuations around this stable scaffold are expressions of the actual physiological brain state.

However, the brain develops during the lifespan, and particularly during childhood and adolescence, it suffers crucial structural changes. Thus, the question remains whether a distinct stationary pattern of relationships encompassing the entire scalp is also found in children as in adult subjects, and second, whether the SCP based on functional relationships also undergoes changes such as those in the structural reorganization of brain networks. Furthermore, it might be interesting to prove whether also in children cognitive brain states could be better described and characterized by deviations from the SCP.

Some hints in favor of this hypothesis can be found in the literature. Generally, changes in frequency bands amplitude have been described in children while they are at rest and during the performance of cognitive tasks (i.e. Benninger et al., 1984; Marshall et al., 2002; Clarke et al., 2001; Perone et al., 2018). Concerning EEG functional connectivity maturation, some studies have been conducted in the resting state. In an early report, Marosi et al. (1992) found an increase in EEG coherence with higher age. In another study (Thatcher et al., 2008), authors observed maturational changes in intra-hemispheric coherence in children and adolescents aged 6 months to 16 years. That study reported large changes in EEG coherence and phase in children aged 6 months to 4 years, followed by a significant linear trend towards higher coherence in short inter-electrode distances, and longer phase delays in long inter-electrode distances. Along this line, Fair et al. (2009) observed that functional brain development proceeds from a local to a distributed communication organization. It is worth noting that disruptions in EEG functional connectivity have been described in different disorders such as attentional deficit-hyperactivity disorders (Barry et al., 2002; Murias et al., 2007), autism spectrum disorder (Kikuchi et al., 2013), and intellectual disability (Gasser et al., 1987).

However, only sparse data exist that addressed EEG functional connectivity during cognitive processing in typically-developing children. Machinskaya and Kurganskiĭ (2012) compared coherent activity between children (7-8 years old) and adults during a working memory task. While they reported for the adult group, an increase of the coherence in the alpha band between frontoparietal regions, predominantly in the right hemisphere, in children they detect significant coherence values in the inferior-temporal and parietal cortical regions coherence in the theta band. These results are interpreted in terms of a relative immaturity of the mechanisms of executive control of working memory in children. In adults, variations in functional connectivity during the processing of diverse tasks include global and specific network changes associated with information processing (i.e. Daume et al., 2017; Hearne et al., 2017; Maurits et al., 2006; Vatansever et al., 2017).

In the present study, we were interested in exploring the developmental changes during attentional processes using high salient emotional stimuli such as facial expressions, due to their relevance in social interaction and adaptation (Dekowska et al., 2008). As Driver and Frackowiak (2001) state, selective attention allows people to process some stimuli more thoroughly than others, which is partly under voluntary control, and partly determined by stimulus salience. Like other cognitive abilities, facial and emotional recognition improves with age in school-age children. For example, Damaskinou and Watling (2018) presented evidence that 10-year-olds overperform 6-year-olds on emotional recognition tasks.

One of the most widely used paradigms for studying brain electrical responses originated during facial emotional recognition is the odd-ball task. Fichtenholtz et al. (2007) propose that the odd-ball task may be useful to study the interaction of the attention and emotion processing. In this paradigm, the participant is asked to respond to a specific low-probability target stimulus which is presented within a stream of high-probability non-target stimuli.

Some EEG and MEG studies employing several analysis approaches have achieved to find functional connectivity among the face-sensitive brain areas of the ventral visual pathway that includes primary occipital regions, the inferior-temporal cortex, and especially the fusiform face area (Yang et al., 2015; Maffei & Sessa, 2021; Yin et al., 2021). However, little is known about developmental changes in functional connectivity during cognitive activity, particularly during face processing. Cohen Kadosh et al. (2011) examined the fMRI connectivity of the core face network and observed that it develops during childhood. However, children did not show the modulation in the functional network connections by task demands found in adults.

A recent fMRI study (Harrewijn et al., 2021) was conducted to test the similarity in functional connectivity between rest and while performing a dot-probe task with neutral, happy, and angry faces in 13 years-old children. Results revealed that functional connectivity during rest and a dot-probe task was positively correlated and that the similarity levels were related to threat bias. In another study in adults, Yin et al. (2021) found that during face processing, the EEG brain network was more efficient for information transfer and exchange compared with non-face processing.

To our knowledge, the presence of a SCP in EEG activity has not been studied in children so far, neither at rest nor during task performance. Studying EEG functional connectivity in typically-developing children may shed light on the normal maturational changes in the brain dynamics organization while performing a cognitive task with salient stimuli (faces) across ages and it may provide a basis for the understanding of functional abnormalities in special populations. Finally, the estimation of variations from the stationary correlation patterns may provide differentiation of dynamical transient changes during different cognitive states in children like those observed in adult subjects (Olguín-Rodríguez et al., 2018). Therefore, the aims of the present study were threefold:

1) To identify a possible SCP in children similar to that found in adults;
2) To evaluate the global variations *via* similarity estimates between SCP and the correlation pattern of each condition during an odd-ball task with facial stimuli.
3) To identify the effects of age *via* possible changes of the coupling between brain regions, during the performance of two tasks that requires attention and face processing.

We hypothesized that a correlation pattern like that found in adults would exist in children. The similarity metric will show lower values during cognitive activity than during resting state, indicating higher transient dynamical features. In addition, we predicted that cross-correlations would increase with age and will be modulated by task demands.

## Methods

### Participants

The sample consisted of 64 right-handed children, distributed into three age groups, each with a range of 11 months (G1: 10 boys and seven girls, mean age = 6.30, SD = 0.44; G2: 13 boys and nine girls, mean age = 8.31, SD = 0.37 and; G3: 15 boys and 10 girls, mean age = 10.2, SD = 0.45). The children group were homogeneous with respect to their age and school grade and normal IQ scores. None had any history of neurological disorders. The parents provided their written informed consent for their children´s participation in this study. The project was approved by the Ethics Committee of the Institute of Neurosciences following the Declaration of Helsinki.

### EEG recording

EEG was continuously recorded during the experimental conditions: at rest with eyes opened and during task performance in the leads F3, F4, Fz, C3, C4, Cz, T3, T4, T5, T6, P3, P4, Pz, O1, and O2 according to the 10/20 International System with linked earlobes as a reference with a sampling rate of 500 Hz. To this end, we used a Medicid five acquisition device. Electrode impedances were assured to be less than 5 kOhms. To control artifacts caused by ocular movements an electrooculogram was recorded simultaneously *via* two electrodes located at the extreme upper corner of the right eye and the lower outer edge of the left eye, respectively. During acquisition, the signal was bandpass filtered between 0.01 and 50 Hz.

### Experimental design

An oddball paradigm was used for the presentation of two tasks. In one of them, the children had to recognize the facial expressions of happiness; and in another one, they had to identify the sex of the models. For each task, a total of 200 stimuli were randomly presented, of which 70% were non-target frequent stimuli and the remaining 30% were the target infrequent ones. Each trial started with a fixation point (a white cross) in the center of the screen with a variable duration between 800 and 1,300 ms, followed by either a frequent or a target stimulus presented for 700 ms.

Participants were asked to respond to the target stimuli by pressing a key. For the happiness condition, the target stimuli were faces with a happiness expression, while the non-target stimuli consisted of men´s and women´s faces with a neutral expression. For the sex recognition task, the target stimuli were female faces and the non-target male ones.

### Procedure

Once the presence of the inclusion criteria for each participant were determined, we asked the child to sit 60 cm away in front of the computer screen and perform the task, avoiding eye and head movements while the faces were shown. We counterbalanced the presentation of the two tasks among participants and allowed a 5-min rest period between them. Before starting each task, participants underwent a training block of 10 trials to assure that they have completely understood the instructions.

### EEG analysis

After visual inspection, data preprocessing was conducted applying Independent Components Analysis (ICA) to remove eye movements (Delorme & Makeig, 2004). On the average we used 25 artifacts free EEG segments that corresponded exclusively to correct responses to the targets which were considered for further analysis. Anterior-temporal electrodes (T3 and T4) were excluded because signals were contaminated with artifacts in some children and these regions are not directly involved in face identification or attentional processes. The computer codes for the numerical analysis were elaborated using MATLAB.

Continuous EEG signals were first segmented into windows of T = 1 s length. In the case of the tasks, this window began in synchrony with the stimulus presentation. We then, filtered the signals by a fourth-order Butterworth filter (1-25 Hz). We calculated for each segment the Pearson’s correlation coefficients, *viz.* zero-lag cross-correlations,

$$C_{ij}=\frac{1}{T}\overset{T}{\underset{k=1}{\sum }}X_{i}\left(t_{k}\right)X_{j}\left(t_{k}\right)$$

between each pair of electrodes, adapting the procedure followed in previous studies (Arzate-Mena et al., 2022; Müller et al., 2014; Olguín-Rodríguez et al., 2018). Here $C_{ij}$ denotes the cross-correlation matrix of two time series $X_{i}\left(t_{k}\right){\;X}_{j}\left(t_{k}\right)$ , *T* denotes the number of samples of the data segment (*i,j =* 1, … , *N* and *k =* 1, … *T*) and N the number of electrodes. In this formula, the data $X_{i}\left(t_{k}\right){\;X}_{j}\left(t_{k}\right)$ are normalized to zero mean and unit variance, such that the correlation coefficient takes values between ±1 . The resulting matrix is real-symmetric and all diagonal elements are equal to one. Then we estimated the individual stable correlation pattern (SCP) by averaging all the correlation matrices of all conditions (resting state and tasks) for each participant.

To obtain the similarity metric, we calculated the correlation values (Pearson´s analysis) between the SCP and the correlation matrix of each EEG segment based on the procedure used by Olguín-Rodríguez et al. (2018) and Arzate-Mena et al. (2022). Thereby, we ordered the triangle of each matrix in a vector and normalized its elements to zero mean unit variance to ensure that Pearson coefficients take values between one and minus one. Then we estimated the Pearson coefficients between these vectors. Note, lower similarity indicates larger topological changes of the functional network with respect to the average. These changes, viz deviations from the SCP might be characteristic for the condition under consideration.

### Statistical analysis

For testing behavioral differences in the number of correct responses and reaction times between conditions as a function of age, we applied mixed ANOVAs (age x conditions). The SCP correlation coefficients were compared among age groups through a Welch´s *t*-test. In addition, we applied a mixed ANOVA (age x conditions) to evaluate similarity metric differences. Greenhouse corrections were applied when necessary and Bonferroni corrections for multiple testing of pairwise comparisons. A *p-*value less than 0.05 was considered for significant differences.

To observe an overall effect of age and conditions, we calculated the average difference (task–rest) of the correlation matrices for sex and happiness, and their empirical cumulative distribution functions using the elements below the main diagonal. Then, a two-sample Kolmogorov-Smirnov (K-S) test was applied to determine the probability that two samples derived for the different age-groups (6 vs. 8, 8 vs. 10, and 6 vs. 10 years) stem from the same probability distribution. We illustrate our results of considerable changes of the average cross-correlation matrices like a network upon the scalp, where “considerable” means that the changes surpass a threshold of one standard deviation above or below the SCP-value.

## Results

### Behavioral results

At the behavioral level, the percentage of correct responses increased and the reaction times decreased with higher age (Table 1). However, differences were apparent between the two tasks, with the sex-based one showing greater difficulty. A significant interaction of age X conditions (*F*_*(2,61)*_ *= 5.43, p = 0.01,* $\eta^{2}$ *= 0.15*) revealed that the accuracy rate was lowest in the youngest group in relation to the other two groups on the sex task (*p* < 0.01), and in relation to the 10-years-olds on the happiness task. The sex task showed lower accuracy than the happiness one in all groups (*p* < 0.01).

**TABLE 1:** Percentage of correct responses and the reaction times in each age group (6, 8 and 10 years old).

	Correct responses		Reaction times	
	Sex Mean (SD)	Happiness Mean (SD)	Sex Mean (SD)	Happiness Mean (SD)
6	50.68 (17.52)	76.27 (12.11)	766.98 (73.46)	723.27 (79.24)
8	70.15 (19.75)	80.83 (15.91)	681.76 (95.04)	659.31 (58.75)
10	76.27 (12.20)	90.67 (7.33)	647.42 (55.78)	619.17 (56.50)

Reaction times to the target stimuli showed significant differences for both age (*F*_*(2,61)*_ *= 17.69, p = 0.001*, $\eta^{2}$ *= 0.36*) and conditions (*F*_*(1,61)*_ *= 11.72, p = 0.01,* $\eta^{2}$ *= 0.16*). Reaction times were shorter in the older groups than in the group of 6-years-old (*p* < 0.001), and shorter for the happiness than the sex condition (*p* < 0.001).

### Stable correlation pattern

**FIGURE 1:**
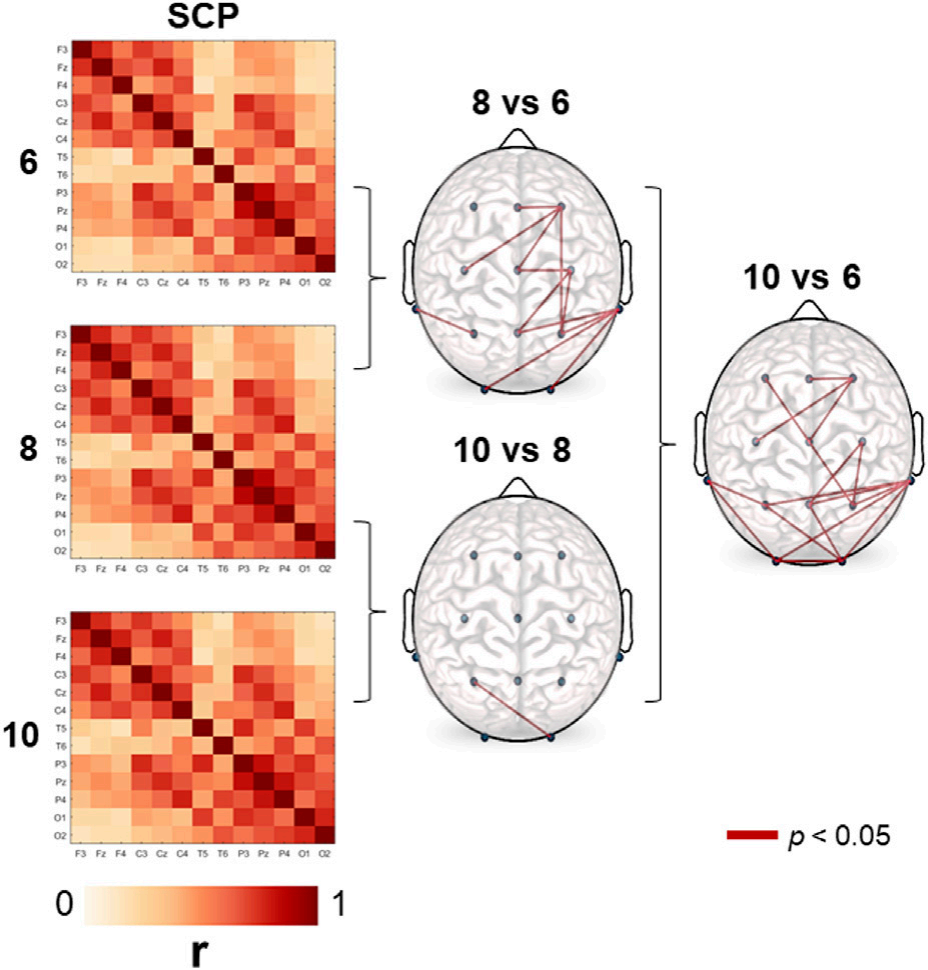
Stable correlation pattern (SCP) for each age group. Connection lines indicate the age-significant differences (6, 8, and 10-year-old) in cross-correlation values on a 5% significance level according to the *t*-test. Correlation estimates turn out to be systematically higher in the older group in all comparisons.

As it can be observed in Figure 1, the structure of the average correlation matrices is very similar across the age groups. However, some differences are observable among the age groups. Systematically, we observed that correlations increase with age. In the graph on the right-hand side of Figure 1 we show the correlation coefficients of between those electrode pairs that resulted significant on a 5%-level according to the *t*-test. The differences were basically seen in a longitudinal arranged network that includes frontal, central, and parietal regions, and another posterior network that includes posterior-temporal, parietal, and occipital regions.

Note, the SCP obtained in the present study is qualitatively different from those presented in Müller et al. (2014) and Olguín-Rodríguez et al. (2018). This striking difference is due to the different reference schemes used. While in those studies the median reference has been chosen, in the present work we used here linked earlobes. As outlined in Rios-Herrera et al. (2019), the earlobe reference may induce redundant information to all data channels, which provokes elevated correlations between all electrode pairs. However, the fact that we obtained also a pronounced stationary correlation pattern using this reference scheme, substantiates the argumentation expressed in those studies (Müller et al., 2014; Olguín-Rodríguez et al., 2018).

### Similarity between the stable pattern and the correlation matrices averaged separately for each condition

**FIGURE 2:**
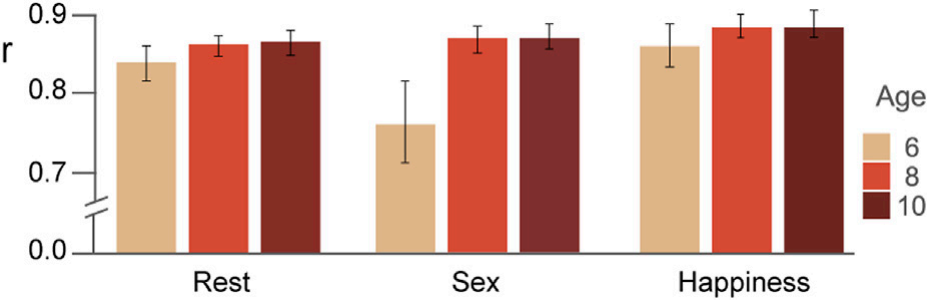
Similarity estimated *via* Pearson correlations (mean and standard errors) between the stable pattern and the average correlation matrix of each condition and for each age group are displayed.

In Figure 2, the Pearson coefficients for the comparison of the SCP and the correlation matrix averaged separately for each condition are displayed for each age group. The ANOVA revealed significant differences for the factors of age (*F*_*(2,61)*_ *= 10,41, p = 0.01*, $\eta^{2}$ = 0.25), conditions (*F*_*(4,122)*_ *= 38.87, p = 0.*01, $\eta^{2}$ = *0.38*) and for interaction between them (*F*_*(4,122)*_ *= 8.77, p = 0.0*1, $\eta^{2}$ *= 0.22*). We observed lower similarity metric values in the youngest children compared to the eight- and 10-year-old groups in the sex condition (*p* < 0.01).

### Rest-to-task cumulative distributions

**FIGURE 3:**
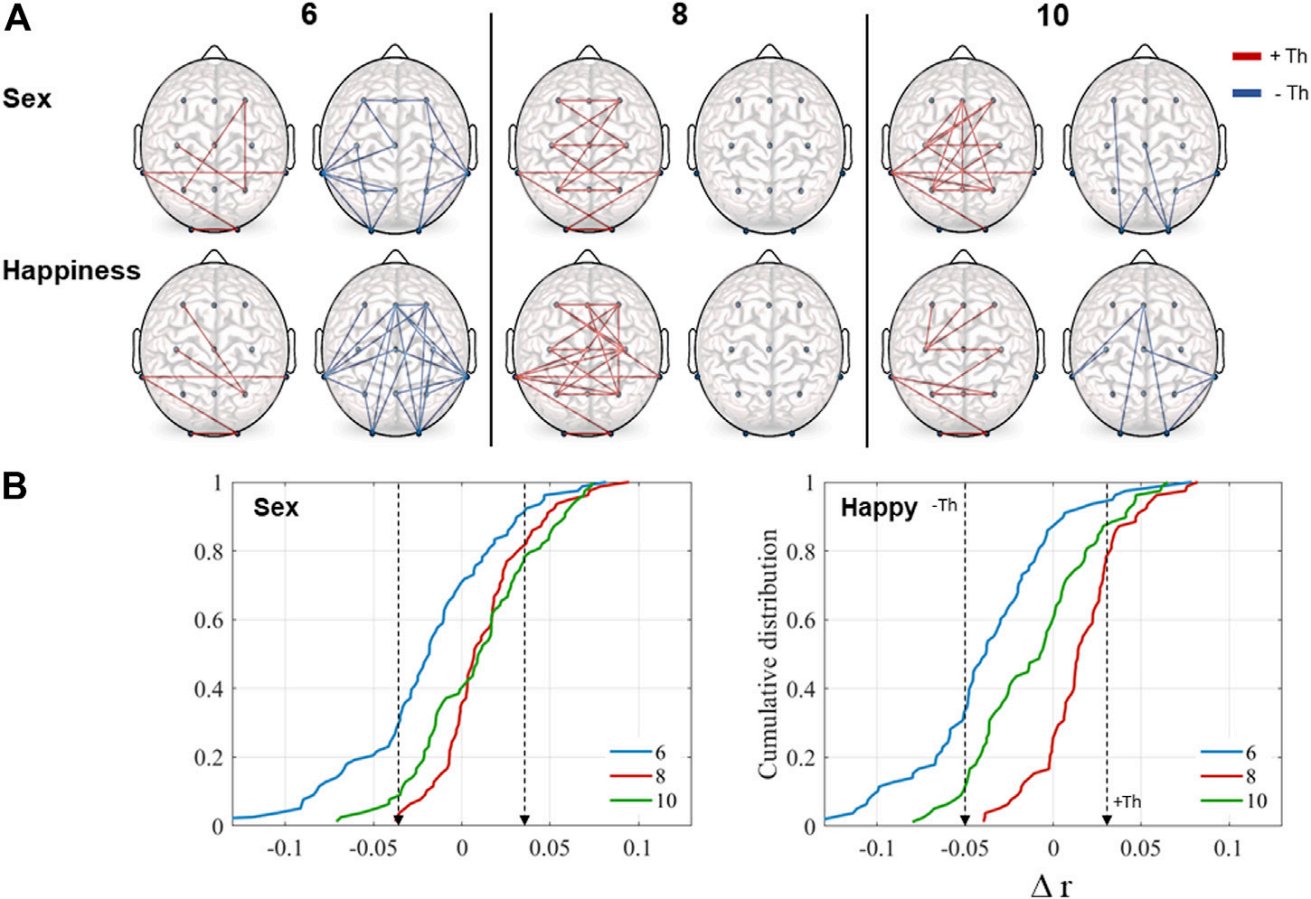
**(A)** Representation of task-to-rest subtraction in correlation values ( ∆r) . Edges are traced when ∆r surpassed the positive and negative thresholds. Red lines indicate an increase in correlation values and the negative ones a decrease. For sex condition: +Th=0.04 and −Th=−0.04 . For happiness: +Th=0.03 and −Th=−0.05 . **(B)** Age differences in cumulative distribution curves of task-to-rest subtraction for each task. Dotted arrows indicate the thresholds used for tracing the graphs.

Figure 3A illustrates rest-to-task subtraction in correlation values for the sex and happiness conditions according to the thresholds obtained by the cumulative distributions functions including the three age groups. Regions that showed an increment (red), as well as those which showed a decrement (blue) during performance of each task in relation to the resting state, are presented. As it can be observed, in the 6-year-olds group, an increase in synchronization between some electrode pairs occurred but there was also a decrease between several pairs in both conditions. In the 8-year-olds group, there was only an increase in correlation, on one hand, between pairs of those regions related to the attentional network (frontal, central, and parietal), and on the other, those related to the face core network (posterior-temporal, parietal and occipital). Finally, in the oldest group, there was also an increase between some of the same regions, mainly in the sex condition, adding frontal-parietal connections. As well, there was a decrease between some long-distance connections including occipital regions.

The K-S test revealed significant differences among the age groups in the cumulative distributions. In general, all distributions were statistically different (Figure 3B). Differences were found to be significant for the sex condition (6 vs. 8, *D(78) = 0.48, p = 0.001*; 8 vs. 10, *D(78) = 0.21, p = 0.04*; 6 vs. 10, *D(78) = 0.32, p = 0.001*) and in the happiness condition (6 vs. 8, *D(78) = 0.7, p = 0.001*; 8 vs. 10, *D(78) = 0.41, p = 0.001*; 6 vs. 10, *D(78) = 0.33, p = 0.001*). On both tasks, the 6-years-old children had negative values, indicating lower correlation values during the tasks compared to the resting condition. In contrast, results for the groups of eight- and 10-years-old displayed positive values, indicating increased correlation.

## Discussion

We first verified the existence of a stable stationary correlation pattern (SCP) in children aged 6-10 years. Functional connectivity increased with age mainly in a longitudinal network that included frontal, central, and parietal regions, but also in a posterior network comprised of posterior-temporal, parietal, and occipital regions. The similarity coupling of the SCP with each condition pattern showed age-dependent changes as a function of task demand. Overall, the youngest children showed a lower correlation value while executing the sex task.

Children displayed a similar SCP to that observed previously in healthy adults in different physiological states -wakefulness and sleep stages-as well as with epilepsy using the same analytical approach. In addition, pronounced correlations between distant electrode pairs has been found using the median EEG-reference, but then with negative sign (Arzate-Mena et al., 2022; Müeller et al., 2014; Olguín-Rodríguez et al., 2018). The finding of an SCP indicates that children´s brains exhibit a stable functional organization pattern from an early age, while also highlighting the importance of spontaneous brain activity in the resting state where, though not related to a known synchronized event, diverse cognitive activity is occurring. Since similarities between structural and functional connectivity have been described (Suárez et al., 2020), and the structural pattern is established early in development (Fan et al., 2011), one could expect a stable functional pattern to emerge in early childhood. Based on their study of brain connectivity in five- and 7-year-olds in the resting-state, Boersma et al. (2011) posited that a shift from random to more organized small-world functional networks characterize normal brain maturation.

Although the SCP was quite similar among the three groups, age-dependent differences appeared. Overall, correlation values from the SCP increased with age between electrode pairs corresponding primarily to regions related to the attentional network (frontal, central and parietal), and those related to the face network (posterior-temporal, parietal and occipital). The differences between the six- and 8-year-olds were clearly lateralized in the right hemisphere. Similar differences were observed between the six- and 10-year-olds but with a more local increase in the attentional network, and between regions related to the face network. The structure of these age pattern changes shows a relation to tasks type (odd-ball) and stimuli (faces) used. On a working memory task, Baum et al. (2019) described an increase of the structural-functional coupling, primarily in the rostrolateral prefrontal cortex across development (8-23 years), that showed higher inter-individual variability during task performance than at rest. Shirer et al. (2011) observed shifting patterns of fMRI connectivity associated with distinct cognitive states in adults.

Our results partially concur with other studies which found that EEG coherence within hemisphere regions increases as a function of age (Marosi et al., 1992; Barry et al., 2004; Thatcher et al., 2008). The increase in functional connectivity may be partially due to the increase of anatomical connections in the corpus callosum and other fibers and its myelination, which have been associated with the maturation of various cognitive functions (Hagmann et al., 2010; Tanaka-Arakawa et al., 2015). In this regard, the maturation of the corpus callosum and other cortical connections may form anatomical substrates that facilitate information transfer during task performance in older children. Fair et al. (2008) found that a similar default network to the one in adults is only sparsely connected in children aged seven to nine, and that there is a continuous increase in correlation strength over age between long-range connections of the network.

The similarity coupling analysis revealed global changes in the stability of the SCP and the changes that emerged from specific ongoing activity during execution of the odd-ball tasks. The youngest children showed a marked decrease in the similarity metric in the sex condition that did not happen in the other two groups. It is noteworthy that the sex task proved to be more difficult than the happiness one, with the difficulty residing in the perceptual features of the faces presented, since all the clues that facilitate identifying sex -hair and make-up- were removed, leaving only the face contour. Apparently, the youngest children had not completely developed the abilities required to process specific identifying features of faces. In contrast, happiness expressions are the ones recognized best and more quickly and precisely from childhood through adulthood (Juth et al., 2005; Mancini et al., 2013; Chronaki et al., 2015; Brechet, 2017) and may be processed using a more holistic strategy. Another point to consider is that happiness is the emotion that most attracts people´s attention (Becker et al., 2011), so it could help improve performance on odd-ball tasks. Hearne et al. (2017) described that although the increment in task complexity did not change the established modular architecture, it did affect selective patterns of connectivity among frontoparietal, subcortical, cingulo-opercular, and default-mode networks. Larger increases in network efficiency within the newly established task modules were associated with higher reasoning accuracy. Our results partially agree with those obtained by Harrewijn et al. (2021) with fMRI, as they determined that functional connectivity patterns during rest and while executing a dot-probe task -with neutral, happy, and angry faces-were positively correlated, and that the similarity levels in 13 years-old children were primarily related to threat bias.

Some fMRI studies, conducted in adults, mention the relationship between the resting-state and active cognitive patterns. Smith et al. (2009) demonstrated that the functional networks utilized by a brain in action are continuously and dynamically active even when subjects are at rest. In their work, Cole et al. (2014) identified a whole-brain network architecture across different tasks that was quite similar to the resting-state network architecture, suggesting an intrinsic, standard architecture of functional brain organization. However, the task-general network architecture was able to distinguish task states from the resting condition. Based on the foregoing, we propose that the similarity metric is a global brain connectivity index that is appropriate for estimating age-related and dynamic changes according to task demand.

Differences across ages related to task demands were also visible in the cumulative distributions. The 6-years-old children had negative values on both tasks, indicating lower correlation values during the tasks compared to the resting state. The two older groups, in contrast, showed mainly positive values, indicating enlarged correlation. As it can be seen in the rest-to-task subtractions, network changes indicate that an increase in synchronization between some electrode pairs occurred in the 6-year-old-group, accompanied by a decrease between several pairs in both conditions. The correlation decrements displayed a different pattern between tasks, though. The 8-year-old group only showed an increase in correlation, on the one hand, between pairs of regions related to the attentional network (frontal, central, and parietal), and on the other, those related to the face core network (posterior-temporal, parietal and occipital). Finally, the oldest group showed an increase between some of the same regions, mainly in the sex condition, but added frontal-parietal connections. There was a decrease between some long-distance connections, including occipital regions. Miskovic et al. (2015) observed that the oldest age group in their work (11-years-old) exhibited the densest patterns of EEG functional connectivity across distant cortical regions, specifically in the alpha band. Those findings are broadly consistent with one of the more replicable fMRI results involving a trend toward increased integration among distant neural networks (Betzel et al., 2014; Fair et al., 2008; 2009; Supekar et al., 2009). In an fMRI study, Brázdil et al. (2007) found a bidirectional information flow between frontal and parietal regions, mainly in the right hemisphere, involved in attentional processing during an odd-ball task. Those regions are also some of the principal structures considered in the generation of P3b, which has been implicated in the closure of the cognitive event encoding cycle (Halgren, et al., 1998).

With respect to the core face network, Cohen Kadosh et al. (2011) examined the fMRI connectivity during identity and happiness task, observing that although the overall structure of the final mature network was present in 7 years-old children, it develops across childhood. The children in that study, however, did not show the modulation in the functional network connections by task demands that was seen in adults. The authors suggested that the emergence of the face network is due to continuous specialization and fine-tuning within the regions of this network.

While most previous research has analyzed resting-state EEG functional connectivity, we observed changes during task performance that required the involvement of various processes and the activation of underlying neural networks. Briefly, our results support the notion that EEG functional connectivity accounts for age-related developmental changes in cognitive abilities related to processes of attention and face identification.

Our study does, however, presents some limitations, first, the small sample size. Second, we used only the 10/20 System montage, including a few electrodes associated to the main regions involved in odd-ball task performance. Further studies could address changes in all frequency bands, since some studies have found major changes in functional connectivity in the theta and alpha bands during task performance. Moreover, the study of sex differences in functional connectivity across ages would be desirable, since there is evidence of different EEG patterns in both sexes in adults, and during the resting stage in children.

## Conclusion

Findings from this study suggest that a base EEG functional network pattern exists from early childhood, which reorganizes across child development. Moreover, functional connectivity is modulated to dynamically adapt to the demands of information processing. The similarity metric may represent an index of global brain connectivity that could be useful in estimating age- and task-related changes. Rest-to-task correlation variations could indicate that the older children in our study generated more efficient coupling of the areas related to the attentional- and face networks, which may underlie the improvement performance they achieved. The study of EEG functional connectivity, therefore, seems to offer a promising approach to discerning maturational changes during the development of diverse cognitive processes and to our understanding of functional disorders in clinical populations.

## Data Availability

The raw data supporting the conclusion of this article will be made available by the authors upon reasonable request.

## References

[B1] Ansari-Asl K. Senhadji L. Bellanger J. J. Wendling F. (2006). Quantitative evaluation of linear and nonlinear methods characterizing interdependencies between brain signals. Phys. Rev. E Stat. Nonlin. Soft Matter Phys. 74 (3), 031916. 10.1103/PhysRevE.74.031916 17025676 PMC2071949

[B2] Arzate-Mena J. D. Abela E. Olguín-Rodríguez P. V. Ríos-Herrera W. Alcauter S. Schindler K. (2022). Stationary EEG pattern relates to large-scale resting state networks–An EEG-fMRI study connecting brain networks across time-scales. NeuroImage 246, 118763. 10.1016/j.neuroimage.2021.118763 34863961

[B3] Bakhshayesh H. Fitzgibbon S. P. Janani A. S. Grummett T. S. Pope K. J. (2019). Detecting connectivity in EEG: A comparative study of data-driven effective connectivity measures. Comp. Bio. Med. 105, 1–16. 10.1016/j.compbiomed.2019.103329 31425938

[B4] Barry R. J. Clarke A. R. McCarthy R. Selikowitz M. (2002). EEG coherence in attention-deficit/hyperactivity disorder: A comparative study of two DSM-IV types. Clin. Neurophysiol. 113 (4), 579–585. 10.1016/S1388-2457(02)00036-6 11956003

[B5] Barry R. J. Clarke A. R. McCarthy R. Selikowitz M. Johnstone S. J. Rushby J. A. (2004). Age and gender effects in EEG coherence: I. Developmental trends in normal children. Clin. Neurophysiol. 115 (10), 2252–2258. 10.1016/j.clinph.2004.05.004 15351366

[B6] Baum G. L. Cui Z. Roalf D. R. Ciric R. Betzel R. F. Larsen B. (2019). Development of structure–function coupling in human brain networks during youth. Proc. Natl. Acad. Sci. U. S. A. 117 (1), 771–778. 10.1073/pnas.1912034117 31874926 PMC6955327

[B7] Becker R. Reinacher M. Freyer F. Villringer A. Ritter P. (2011). How ongoing neuronal oscillations account for evoked fMRI variability. J. Neurosci. 31, 11016–11027. 10.1523/JNEUROSCI.0210-11.2011 21795550 PMC6623098

[B8] Benninger C. Matthis P. Scheffner D. (1984). EEG development of healthy boys and girls. Results of a longitudinal study. Electroencephalogr. Clin. Neurophysiol. 57 (1), 1–12. 10.1016/0013-4694(84)90002-6 6198138

[B9] Betzel R. F. Byrge L. He Y. Goñi J. Zuo X. N. Sporns O. (2014). Changes in structural and functional connectivity among resting-state networks across the human lifespan. Neuroimage 102, 345–357. 10.1016/j.neuroimage.2014.07.067 25109530

[B10] Boccaletti S. Latora V. Moreno Y. Chavez M. Hwang D. U. (2006). Complex networks: Structure and dynamics. Phys. Rep. 424 (4-5), 175–308. 10.1016/j.physrep.2005.10.009

[B11] Boersma M. Smit D. J. A. de Bie H. M. A. Van Baal G. C. M. Boomsma D. I. de Geus E. J. C. (2011). Network analysis of resting state EEG in the developing young brain: Structure comes with maturation. Hum. Brain Mapp. 32, 413–425. 10.1002/hbm.21030 20589941 PMC6870229

[B12] Brázdil M. Mikl M. Mareček R. Krupa P. Rektor I. (2007). Effective connectivity in target stimulus processing: A dynamic causal modeling study of visual oddball task. Neuroimage 35 (2), 827–835. 10.1016/j.neuroimage.2006.12.020 17258910

[B13] Brechet C. (2017). Children's recognition of emotional facial expressions through photographs and drawings. J. Genet. Psychol. 178 (2), 139–146. 10.1080/00221325.2017.1286630 28323534

[B14] Bullmore E. Sporns O. (2009). Complex brain networks: Graph theoretical analysis of structural and functional systems. Nat. Rev. Neurosci. 10, 186–198. 10.1038/nrn2575 19190637

[B15] Chronaki G. Hadwin J. A. Garner M. Maurage P. Sonuga‐Barke E. J. (2015). The development of emotion recognition from facial expressions and non‐linguistic vocalizations during childhood. Br. J. Dev. Psychol. 33 (2), 218–236. 10.1111/bjdp.12075 25492258

[B16] Chu C. J. Tanaka N. Diaz J. Edlow B. L. Wu O. Hämäläinen M. (2015). EEG functional connectivity is partially predicted by underlying white matter connectivity. Neuroimage 108, 23–33. 10.1016/j.neuroimage.2014.12.033 25534110 PMC4323839

[B17] Clarke A. R. Barry R. J. McCarthy R. Selikowitz M. (2001). Age and sex effects in the EEG: Development of the normal child. Clin. Neurophysiol. 112 (5), 806–814. 10.1016/S1388-2457(01)00488-6 11336896

[B18] Cohen J. R. D'Esposito M. (2016). The segregation and integration of distinct brain networks and their relationship to cognition. J. Neurosci. 36 (48), 12083–12094. 10.1523/JNEUROSCI.2965-15.2016 27903719 PMC5148214

[B19] Cohen Kadosh K. Cohen Kadosh R. Dick F. Johnson M. H. (2011). Developmental changes in effective connectivity in the emerging core face network. Cereb. Cortex 21 (6), 1389–1394. 10.1093/cercor/bhq215 21045001 PMC3094719

[B20] Cole M. W. Bassett D. S. Power J. D. Braver T. S. Petersen S. E. (2014). Intrinsic and task-evoked network architectures of the human brain. Neuron 83 (1), 238–251. 10.1016/j.neuron.2014.05.014 24991964 PMC4082806

[B21] Corsi-Cabrera M. Galindo-Vilchis L. Del-Río-Portilla Y. Arce C. Ramos-Loyo J. (2007). Within-subject reliability and inter-session stability of EEG power and coherent activity in women evaluated monthly over nine months. Clin. Neurophysiol. 118 (1), 9–21. 10.1016/j.clinph.2006.08.013 17055781

[B22] Corsi-Cabrera M. Solis-Ortiz S. Guevara M. A. (1997). Stability of EEG inter-and intrahemispheric correlation in women. Electroencephalogr. Clin. Neurophysiol. 102 (3), 248–255. 10.1016/S0013-4694(96)95179-6 9129580

[B23] Damaskinou N. Watling D. (2018). Neurophysiological evidence (ERPs) for hemispheric processing of facial expressions of emotions: Evidence from whole face and chimeric face stimuli. Laterality 23 (3), 318–343. 10.1080/1357650X.2017.1361963 28857672

[B24] Daume J. Gruber T. Engel A. K. Friese U. (2017). Phase-amplitude coupling and long-range phase synchronization reveal frontotemporal interactions during visual working memory. J. Neurosci. 37 (2), 313–322. 10.1523/JNEUROSCI.2130-16.2016 28077711 PMC6596581

[B25] Dekowska M. Kuniecki M. Jaśkowski P. (2008). Facing facts: Neuronal mechanisms of face perception. Acta Neurobiol. Exp. 68 (2), 229–252. 10.55782/ane-2008-1692 18511959

[B26] Delorme A. Makeig S. (2004). Eeglab: An open source toolbox for analysis of single-trial EEG dynamics including independent component analysis. J. Neurosci. Methods 134 (1), 9–21. 10.1016/j.jneumeth.2003.10.009 15102499

[B27] Driver J. Frackowiak R. S. (2001). Neurobiological measures of human selective attention. Neuropsychologia 39 (12), 1257–1262. 10.1016/S0028-3932(01)00115-4 11566309

[B28] Fair D. A. Cohen A. L. Dosenbach N. U. Church J. A. Miezin F. M. Barch D. M. (2008). The maturing architecture of the brain's default network. Proc. Natl. Acad. Sci. U. S. A. 105, 4028–4032. 10.1073/pnas.0800376105 18322013 PMC2268790

[B29] Fair D. A. Cohen A. L. Power J. D. Dosenbach N. U. Church J. A. Miezin F. M. (2009). Functional brain networks develop from a “local to distributed” organization. PLoS Comput. Biol. 5 (5), e1000381. 10.1371/journal.pcbi.1000381 19412534 PMC2671306

[B30] Fan Y. Shi F. Smith J. K. Lin W. Gilmore J. H. Shen D. (2011). Brain anatomical networks in early human brain development. Neuroimage 54 (3), 1862–1871. 10.1016/j.neuroimage.2010.07.025 20650319 PMC3023885

[B31] Fichtenholtz H. M. Hopfinger J. B. Graham R. Detwiler J. M. LaBar K. S. (2007). Happy and fearful emotion in cues and targets modulate event-related potential indices of gaze-directed attentional orienting. Soc. Cogn. Affect. Neurosci. 2 (4), 323–333. 10.1093/scan/nsm026 18626515 PMC2453519

[B32] Gasser T. Jennen-Steinmetz C. Verleger R. (1987). EEG coherence at rest and during a visual task in two groups of children. Electroencephalogr. Clin. Neurophysiol. 67 (2), 151–158. 10.1016/0013-4694(87)90038-1 2439292

[B33] Hagmann P. Sporns O. Madan N. Cammoun L. Pienaar R. Wedeen V. J. (2010). White matter maturation reshapes structural connectivity in the late developing human brain. Proc. Natl. Acad. Sci. U. S. A. 10744, 19067–19072. 10.1073/pnas.1009073107 PMC2973853 20956328

[B34] Halgren E. Marinkovic K. Chauvel P. (1998). Generators of the late cognitive potentials in auditory and visual oddball tasks. Electroencephalogr. Clin. Neurophysiol. 106 (2), 156–164. 10.1016/S0013-4694(97)00119-3 9741777

[B35] Harrewijn A. Kitt E. R. Abend R. Matsumoto C. Odriozola P. Winkler A. M. (2021). Comparing neural correlates of conditioned inhibition between children with and without anxiety disorders - a preliminary study. Behav. Brain Res. 5, 399. 10.1016/j.bbr.2020.112994 PMC7855938 33160010

[B36] Hearne L. J. Cocchi L. Zalesky A. Mattingley J. B. (2017). Reconfiguration of brain network architectures between resting-state and complexity-dependent cognitive reasoning. J. Neurosci. 37 (35), 8399–8411. 10.1523/JNEUROSCI.0485-17.2017 28760864 PMC6596866

[B37] Juth P. Lundqvist D. Karlsson A. Öhman A. (2005). Looking for foes and friends: Perceptual and emotional factors when finding a face in the crowd. Emotion 5 (4), 379–395. 10.1037/1528-3542.5.4.379 16366743

[B38] Kikuchi M. Shitamichi K. Yoshimura Y. Ueno S. Hiraishi H. Hirosawa T. (2013). Altered brain connectivity in 3-to 7-year-old children with autism spectrum disorder. Neuroimage. Clin. 2, 394–401. 10.1016/j.nicl.2013.03.003 24179793 PMC3777701

[B39] Machinskaya R. I. Kurgansky A. V. (2012). A comparative electrophysiological study of regulatory components of working memory in adults and seven-to eight-year-old children: An analysis of coherence of EEG rhythms. Hum. Physiol. 38 (1), 1–13. 10.1134/S0362119712010136 22567832

[B40] Maffei A. Sessa P. (2021). Event‐related network changes unfold the dynamics of cortical integration during face processing. Psychophysiology 58, 5e13786. 10.1111/psyp.13786 33550632

[B41] Mancini G. Agnoli S. Baldaro B. Ricci Bitti P. E. Surcinelli P. (2013). Facial expressions of emotions: Recognition accuracy and affective reactions during late childhood. J. Psychol. 147 (6), 599–617. 10.1080/00223980.2012.727891 24199514

[B42] Marosi E. Harmony T. Sánchez L. Becker J. Bernal J. Reyes A. (1992). Maturation of the coherence of EEG activity in normal and learning-disabled children. Electroencephalogr. Clin. Neurophysiol. 83 (6), 350–357. 10.1016/0013-4694(92)90070-X 1281080

[B43] Marshall P. J. Bar-Haim Y. Fox N. A. (2002). Development of the EEG from 5 months to 4 years of age. Clin. Neurophysiol. 113 (8), 1199–1208. 10.1016/S1388-2457(02)00163-3 12139998

[B44] Maurits N. M. Scheeringa R. van der Hoeven J. H. de Jong R. (2006). EEG coherence obtained from an auditory oddball task increases with age. J. Clin. Neurophysiol. 23 (5), 395–403. 10.1097/01.wnp.0000219410.97922.4e 17016149

[B45] Miskovic V. Ma X. Chou C. A. Fan M. Owens M. Sayama H. (2015). Developmental changes in spontaneous electrocortical activity and network organization from early to late childhood. Neuroimage 118, 237–247. 10.1016/j.neuroimage.2015.06.013 26057595 PMC4554821

[B46] Mormann F. Fell J. Axmacher N. Weber B. Lehnertz K. Elger C. E. (2005). Phase/amplitude reset and theta–gamma interaction in the human medial temporal lobe during a continuous word recognition memory task. Hippocampus 15 (7), 890–900. 10.1002/hipo.20117 16114010

[B47] Müller M. F. Baier G. Jiménez Y. L. García A. O. M. Rummel C. Schindler K. (2011). Evolution of genuine cross-correlation strength of focal onset seizures., Evolution of genuine cross-correlation strength of focal onset seizures. J. Clin. Neurophysiol. 28 (5), 450–462. 10.1097/WNP.0b013e318231c894 21946370

[B48] Müller M. F. Rummel C. Goodfellow M. Schindler K. (2014). Standing waves as an explanation for generic stationary correlation patterns in noninvasive EEG of focal onset seizures. Brain Connect. 4 (2), 131–144. 10.1089/brain.2013.0192 24494638

[B49] Murias M. Webb S. J. Greenson J. Dawson G. (2007). Resting state cortical connectivity reflected in EEG coherence in individuals with autism. Biol. Psychiatry 62 (3), 270–273. 10.1016/j.biopsych.2006.11.012 17336944 PMC2001237

[B50] Olguín-Rodríguez P. V. Arzate-Mena J. D. Corsi-Cabrera M. Gast H. Marín-García A. Mathis J. (2018). Characteristic fluctuations around stable attractor dynamics extracted from highly nonstationary electroencephalographic recordings. Brain Connect. 8 (8), 457–474. 10.1089/brain.2018.0609 30198323

[B51] Park H. J. Friston K. (2013). Structural and functional brain networks: From connections to cognition. Science 342 (6158), 1238411. 10.1126/science.1238411 24179229

[B52] Pereda E. Quiroga R. Q. Bhattacharya J. (2005). Nonlinear multivariate analysis of neurophysiological signals. Prog. Neurobiol. 77 (1-2), 1–37. 10.1016/j.pneurobio.2005.10.003 16289760

[B53] Perone S. Palanisamy J. Carlson S. M. (2018). Age‐related change in brain rhythms from early to middle childhood: Links to executive function. Dev. Sci. 21, 6e12691. 10.1111/desc.12691 29863816

[B54] Qin J. Chen S. G. Hu D. Zeng L. L. Fan Y. M. Chen X. P. (2015). Predicting individual brain maturity using dynamic functional connectivity. Front. Hum. Neurosci. 16, 9 418. 10.3389/fnhum.2015.00418 PMC4503925 26236224

[B55] Ríos-Herrera W. A. Olguín-Rodríguez P. V. Arzate-Mena J. D. Corsi-Cabrera M. Escalona J. Marín-García A. (2019). The influence of EEG references on the analysis of spatio-temporal interrelation patterns. Front. Neurosci. 941. 10.3389/fnins.2019.00941 PMC6751257 31572110

[B56] Shirer W. R. Ryali S. Rykhlevskaia E. Menon V. Greicius M. D. (2011). Decoding subject-driven cognitive states with whole-brain connectivity patterns. Cereb. Cortex 22 (1), 158–165. 10.1093/cercor/bhr099 21616982 PMC3236795

[B57] Smit D. J. Boersma M. Schnack H. G. Micheloyannis S. Boomsma D. I. Hulshoff Pol H. E. (2012). The brain matures with stronger functional connectivity and decreased randomness of its network. PLoS One 7 (5), e36896. 10.1371/journal.pone.0036896 22615837 PMC3352942

[B58] Smith S. M. Fox P. T. Miller K. L. Glahn D. C. Fox P. M. Mackay C. E. (2009). Correspondence of the brain's functional architecture during activation and rest. Proc. Natl. Acad. Sci. U. S. A. 106, 13040–13045. 10.1073/pnas.0905267106 19620724 PMC2722273

[B59] Suárez L. E. Markello R. D. Betzel R. F. Misic B. (2020). Linking structure and function in macroscale brain networks. Trends Cogn. Sci. 24 (4), 302–315. 10.1016/j.tics.2020.01.008 32160567

[B60] Supekar K. Musen M. Menon V. (2009). Development of large-scale functional brain networks in children. PLoS Biol. 7 (7), e1000157. 10.1371/journal.pbio.1000157 19621066 PMC2705656

[B61] Tanaka-Arakawa M. M. Matsui M. Tanaka C. Uematsu A. Uda S. Miura K. (2015). Developmental changes in the corpus callosum from infancy to early adulthood: A structural magnetic resonance imaging study. PLoS One 10, e0118760. 10.1371/journal.pone.0118760 25790124 PMC4366394

[B62] Thatcher R. W. North D. M. Biver C. J. (2008). Development of cortical connections as measured by EEG coherence and phase delays. Hum. Brain Mapp. 29 (12), 1400–1415. 10.1002/hbm.20474 17957703 PMC6871112

[B63] Vatansever D. Bzdok D. Wang H. T. Mollo G. Sormaz M. Murphy C. (2017). Varieties of semantic cognition revealed through simultaneous decomposition of intrinsic brain connectivity and behaviour. Neuroimage 158, 1–11. 10.1016/j.neuroimage.2017.06.067 28655631

[B64] Yang Y. Qiu Y. Schouten A. C. (2015). Dynamic functional brain connectivity for face perception. Front. Hum. Neurosci. 9, 662. 10.3389/fnhum.2015.00662 26696870 PMC4672064

[B65] Yin Y. Zheng X. Hu B. Zhang Y. Cui X. (2021). EEG emotion recognition using fusion model of graph convolutional neural networks and LSTM. Appl. Soft Comput. 100, 106954. 10.1016/j.asoc.2020.106954

